# Fall Types and Home Hazards: Insights into Risk Factors Among Korean Older Adults

**DOI:** 10.1093/geroni/igaf122.2629

**Published:** 2025-12-31

**Authors:** Gwang Suk Kim, Min Kyung Park, Jae Jun Lee, Layoung Kim, Namhee Kim, Seungbum Yang

**Affiliations:** Yonsei University College of Nursing, Seoul, Republic of Korea; Yonsei University College of Nursing, Seoul, Republic of Korea; Yonsei University College of Nursing, Seoul, Republic of Korea; The University of Suwon, Hwaseong, Republic of Korea; Yonsei University, Wonju, Republic of Korea; Graduate School of Yonsei University, Seoul, Republic of Korea

## Abstract

Most falls occur at home, and individuals with a history of falls are at a higher recurrence risk. This study classified older adults’ fall types and examined the association of each type with fall risk in the home environment. The data of 211 Korean older adults who had experienced falls in the past two years were analyzed. Fall risk in the home environment was assessed using the Revised Korean version of the Home Falls and Accidents Screening Tool, and fall types were identified through latent class analysis (LCA) based on fall location and cause. The highest-risk areas in the home were the bedroom (0.43±0.34), bathroom (0.35±0.14), and stairs (0.27±0.24). The most frequently reported hazards were the absence of grab rails in the bathroom (90%), absence of non-slip mats in shower areas (77.3%), presence of grab rails on only one side of the outdoor stairs (64.9%), and absence of a light switch near the bed (64.0%). The LCA identified four fall clusters: home falls due to loss of balance, home falls due to tripping, outdoor falls, and bathroom falls due to slipping. Fall risk in the home environment varied significantly by fall cluster, being the highest in home falls due to loss of balance and the lowest in bathroom falls due to slipping. Environmental hazards contributing to balance loss and subsequent falls need to be mitigated. Given that bathroom-related hazards were the most frequently reported, slip risk in bathrooms remains high among Korean community-dwelling older adults, regardless of overall home safety.

